# The Role of miRNA in Testicular Cancer: Current Insights and Future Perspectives

**DOI:** 10.3390/medicina59112033

**Published:** 2023-11-17

**Authors:** Francesco Ditonno, Antonio Franco, Celeste Manfredi, Daniela Fasanella, Marco Abate, Roberto La Rocca, Fabio Crocerossa, Vincenzo Iossa, Ugo Giovanni Falagario, Luigi Cirillo, Vincenzo Maria Altieri, Ernesto Di Mauro, Felice Crocetto, Biagio Barone, Simone Cilio, Savio Domenico Pandolfo, Achille Aveta, Vincenzo Mirone, Corrado Aniello Franzese, Davide Arcaniolo, Luigi Napolitano

**Affiliations:** 1 Department of Urology, Rush University Medical Center, Chicago, IL 60612-3833, USA; francesco_ditonno@rush.edu (F.D.); antonio_franco@rush.edu (A.F.); 2 Department of Urology, University of Verona, 37126 Verona, Italy; 3 Department of Urology, Sant’Andrea Hospital, La Sapienza University, 00189 Rome, Italy; 4 Urology Unit, Department of Woman, Child and General and Specialized Surgery, “Luigi Vanvitelli” University, 81100 Naples, Italy; davide.arcaniolo@gmail.com; 5 Department of Life, Health and Environmental Sciences, University of L’Aquila, 67100 L’Aquila, Italy; daniela.fasanella1@graduate.univaq.it; 6 Urology Unit, Department of Neurosciences, Reproductive Sciences and Odontostomatology, University of Naples “Federico II”, 80138 Naples, Italyrobertolarocca87@gmail.com (R.L.R.); cirilloluigi22@gmail.com (L.C.); ernestodm9@gmail.com (E.D.M.); felice.crocetto@gmail.com (F.C.); biagio.barone@unina.it (B.B.); simocilio.av@gmail.com (S.C.); pandolfosavio@gmail.com (S.D.P.); achille-aveta@hotmail.it (A.A.); mirone@unina.it (V.M.); nluigi89@libero.it (L.N.); 7 Department of Urology, Magna Graecia University of Catanzaro, 88100 Catanzaro, Italy; crocerossa@unicz.it; 8 Department of Andrology, “Antonio Cardarelli” Hospital, 80131 Naples, Italy; vincenzoiossa@msn.com; 9 Department of Urology and Organ Transplantation, University of Foggia, 71122 Foggia, Italy; ugofalagario@gmail.com; 10 Department of Medicine and Health Sciences “V. Tiberio”, University of Molise, 86100 Campobasso, Italy; vincenzomaria.altieri@gmail.com; 11 Department of Urology, Humanitas Gavazzeni, 24125 Bergamo, Italy; 12 ASL Napoli 3 Sud, 80121 Naples, Italy; corradofranzese@libero.it

**Keywords:** biomarker, germ cell neoplasia in situ, germ cell tumour, microRNA

## Abstract

*Background and Objectives*: Despite advancements in the diagnosis and treatment of testicular germ cell tumours (TGTCs), challenges persist in identifying reliable biomarkers for early detection and precise disease management. This narrative review addresses the role of microRNAs (miRNAs) as potential diagnostic tools and therapeutic targets in the treatment of TGCTs. *Materials and Methods*: Three databases (PubMed^®^, Web of Science™, and Scopus^®^) were queried for studies investigating the utility of miRNA as diagnostic tools, assessing their prognostic significance, and evaluating their potential to guide TGCT treatment. Different combinations of the following keywords were used, according to a free-text protocol: “miRNA”, “non-coding RNA”, “small RNA”, “Testicular Cancer”, “seminomatous testicular germ cell”, “non-seminomatous testicular germ cell”. *Results*: The potential of miRNAs as possible biomarkers for a non-invasive diagnosis of TGCT is appealing. Their integration into the diagnostic pathway for TGCT patients holds the potential to enhance the discriminative power of conventional serum tumour markers (STMs) and could expedite early diagnosis, given that miRNA overexpression was observed in 50% of GCNIS cases. Among miRNAs, miR-371a-3p stands out with the most promising evidence, suggesting its relevance in the primary diagnosis of TGCT, particularly when conventional STMs offer limited value. Indeed, it demonstrated high specificity (90–99%) and sensitivity (84–89%), with good positive predictive value (97.2%) and negative predictive value (82.7%). Furthermore, a direct relationship between miRNA concentration, disease burden, and treatment response exists, regardless of disease stages. The initial evidence of miRNA decrease in response to surgical treatment and systemic chemotherapy has been further supported by more recent results suggesting the potential utility of this tool not only in evaluating treatment response but also in monitoring residual disease and predicting disease relapse. *Conclusions*: MiRNAs could represent a reliable tool for accurate diagnosis and disease monitoring in the treatment of TGCT, providing more precise tools for early detection and treatment stratification. Nevertheless, well-designed clinical trials and comprehensive long-term data are needed to ensure their translation into effective clinical tools.

## 1. Introduction

Testicular cancer (TC) represents the most frequent tumour in young men between 15 and 45 years, with an incidence of three to ten new cases per 100,000 males/year [1]. Within TC, testicular germ cell tumours (TGCTs) account for over 90–95% of cases, most of which arise from germ cell neoplasia “in situ” (GCNIS) [2]. TGCTs can be classified into seminomas and non-seminomatous germ cell tumours, based on their histological features [3]. Recognised risk factors encompass, among others, cryptorchidism, hypospadias, decreased spermatogenesis, and impaired fertility, which are all components of testicular dysgenesis syndrome [4]. Clinical diagnosis relies on physical examination and the measurement of serum tumour markers (STMs), namely, alpha-fetoprotein (AFP), beta human chorionic gonadotropin (hCG), and lactate dehydrogenase (LDH) [3]. Traditional biomarkers are associated with inherent drawbacks such as low specificity and sensitivity, as well as the potential for elevated levels in other malignancies [5], potentially resulting in false positive or negative results [6]. Due to the limitations of current biomarkers, ongoing research is dedicated to identifying novel, highly sensitive, and specific tools that are easily accessible and minimally invasive, with a quick turnaround time [7]. Micro-RNAs (miRNAs) are endogenous, small, noncoding RNAs physiologically involved in several biological functions, including gene expression and transcription [8]. Additionally, they exert influence on processes such as angiogenesis, energy metabolism, immune system activation, and tumour invasion, making them potential contributors to oncogenesis [9]. Recent findings have highlighted their significance in different tumours, such as lung cancer, several gastrointestinal malignancies, and TGCTs [10,11]. Indeed, the expression of microRNA in TC tissue and their presence in the bloodstream have been associated with the regulation of genes linked to germ cell differentiation and infertility [12]. Therefore, miRNAs could play a pivotal role in the diagnosis, treatment, and prognosis of TGCTs, possibly overcoming the intrinsic limitations of currently available biomarkers. The present study aims to provide a comprehensive overview of the current state of knowledge regarding miRNAs as biomarkers, therapeutic targets, and treatment modulators in patients with TGCTs. The objective was not only to consolidate existing knowledge on the utilisation of miRNA in TGCT but also to pave the way for future advancements in its management and to foster further research in the field.

## 2. Materials and Methods

A narrative review of the literature was conducted to examine the impact of miRNA on the diagnosis and disease management of TGCT. Three databases (PubMed^®^, Web of Science™, and Scopus^®^) were queried for studies investigating the utility of miRNA as diagnostic tools, assessing their prognostic significance, and evaluating their potential to guide TGCT treatment. Different combinations of the following keywords were used, according to a free-text protocol: “miRNA”, “non-coding RNA”, “small RNA”, “Testicular Cancer”, “seminomatous testicular germ cell”, “non-seminomatous testicular germ cell”. Retrospective and prospective studies, both comparative and non-comparative; narrative reviews; systematic reviews; and meta-analysis were included. Reference lists of included studies were searched for additional relevant articles. Letters, editorial comments, replies from authors, case reports, meeting abstracts, and non-human and non-English language articles were excluded. Three senior authors (F.D., C.M., L.N.) selected the most relevant studies for design, sample size and level of evidence, according to their knowledge and experience. Other authors reviewed and approved the study selection. Data were extracted, and the results were qualitatively described, as reported in primary studies.

## 3. Clinical Implications of miRNA

The presence of miRNA in GCNIS and TCGTs tissues has been reported in several studies, leading to the recognition of a subset of miRNA linked to TGCTs: the miR-371-3 cluster, hsa-miR-367-3p, and the miR-302 family [13,14,15]. Moreover, variations in miRNA expression have been observed among different subtypes of TGCTs, with embryonal carcinoma exhibiting the highest levels, teratoma typically showing little to no miRNA expression, and seminoma displaying moderate concentrations [16]. 

### 3.1. Diagnostic Performance

The potential of miRNAs as possible biomarkers for a non-invasive diagnosis of TGCT is appealing. Results from an in-vitro study on 134 tissue samples of patients suffering from TGCT by Vilela-Salgueiro et al. showed how miR-371a-3p concentrations could accurately discriminate TGCTs from normal testicular tissue [17]. Moreover, the presence of an increased concentration of miR-371a-3p in the peripheral blood and hydrocele fluid of TGCT-diagnosed patients provided evidence that this circulating miRNA is specifically produced by malignant cells [18]. 

The clinical applicability of these findings was confirmed in several studies analysing the diagnostic performance of miR-371a-3p, which achieved sensitivities of 84.7%, 88.7%, and 89%, and specificities of 99%, 93.4%, and 90% [19,20,21]. In a prospective multicentre study on 616 patients, Dieckmann et al. reported a specificity of 96.1% for seminoma, a sensitivity of 95% and a specificity of 96.1% for nonseminoma, and an overall positive predictive value (PPV) of 97.2% and a negative predictive value (NPV) of 82.7% with miR-371a-3p [22]. Gillis et al. developed a protocol called the targeted serum miRNA (TSmiR) test, based on magnetic-bead-based purification and qPCR quantification, for the detection of miRNAs in the serum of patients affected by TC. The authors observed a high specificity of miRNA markers since none of them were elevated in the no-TGCT group. The best diagnostic performance was recorded with miR-371-3/367, compared to miR-302abc/200c, leading to a sensitivity of 98% and a specificity of 48.3%. Moreover, miR-371-3/367 outperformed currently available biomarkers in terms of diagnostic performance [23]. In light of these results, Nappi et al. designed a trial aimed at analysing operative characteristics of plasma miR371. An extremely high specificity (100%) and PPV (100%) emerged from this study. On the contrary, data concerning sensitivity and NPV were inconclusive given the short follow-up available [24]. In a case-control prospective study, Badia et al. investigated the relationship between miR-371a-3p in pre-orchiectomy serum and viable TGCT at final pathology. Using an expression threshold of 23.5, sensitivity and specificity reached 93% and 100%, respectively, along with a PPV of 100% and an NPV of 73% [25]. Indeed, a positive correlation between tumour size and the relative expression of elements of the miR-371-3 cluster was reported in a study by Lobo et al., investigating the potential role of miRNA in a liquid biopsy setting [26]. This was particularly true for pure seminoma, in which sensitivity lowered to 77% and 59% for lesions sized <20 mm and <10 mm, respectively. However, sensitivity remained at 100% for non-seminoma lesions <10 mm [27]. 

The implications of this growing body of evidence are severalfold. As a matter of fact, incorporating miRNA measurement into the diagnostic pathway for TGCT patients holds the potential to enhance the discriminative power of conventional STM. Furthermore, it could expedite early diagnosis, given that miRNA overexpression was observed in 50% of GCNIS cases [28]. However, it is essential to note that conflicting evidence regarding the use of miRNA in the initial stages of the disease exists [29]. Therefore, caution is advised when considering the generalisability of these findings. A distinct advantage could be realised in situations where testis-sparing surgery is mandatory, such as monorchid patients [25]. In these scenarios, the current diagnostic tools face challenges in distinguishing between malignant and benign lesions before surgery, and miRNA could help discriminate between these two conditions. Features that make miRNA suitable as a biomarker for TGCT are the high specificity and sensitivity, as well as the short half-life [27]. Among miRNAs, miR-371a-3p stands out with the most promising evidence, suggesting its relevance in the primary diagnosis of TGCT, particularly when conventional STM offer limited value.

However, many in vivo studies did not distinguish between seminoma and non-seminoma patients, making it difficult to definitively evaluate the true potential of miRNA in each histologic subtype. This makes surgical exploration and histological diagnosis unavoidable for a proper diagnosis and management of TC. An effort in terms of future research is needed to establish the correct utilisation of miRNA in the diagnosis of TGCTs according to different histological subtypes.

### 3.2. Treatment Response and Clinical Decision Making

A direct relationship between miRNA concentration and response to treatment exists across different disease stages [30]. Initial evidence of miR-372 decrease in response to systemic chemotherapy [31] has been further supported by more recent results suggesting the potential utility of this tool not only in evaluating treatment response [32] but also in predicting disease relapse [33]. 

#### 3.2.1. Surgical Treatment

While radical orchiectomy represents the backbone of the treatment of TGCT, retroperitoneal lymphadenectomy (RPLND) is still a subject of debate, depending on the final histology and disease stage. Oncological outcomes represent the primary measure of successful treatment; thus, the identification of a liquid biomarker that could predict therapeutic efficacy would be an invaluable tool for clinicians.

A significant decrease in miR-371a-3p levels has been reported after radical orchiectomy in 91.77% and 82.4% of patients with local and metastatic disease, respectively. The authors observed persistent miRNA expression after primary surgical treatment in a small proportion of clinical stage I patients [22]. This phenomenon could be related to occult metastasis, given the high incidence of staging errors reported in TGCTs [32]. Hence, miRNA concentration could be related to tumour bulk and to the presence of viable tumour cells after orchiectomy.

Among the possible management strategies for TGCTs, the role of RPLND has evolved over the years. It currently represents a therapeutic and staging procedure, especially in non-seminomatous TGCTs, but it is widely related to patients’ preferences and expectations. In this clinical scenario, miRNA could help provide more personalised, patient-centred approaches. In a prospective study on 24 chemotherapy-naïve patients undergoing RPLND, miR-371a-3p expression was 13,000 times higher than teratoma or benign tissue, reaching a sensitivity of 100% and a specificity of 92%, making it the most discriminating serum miRNA for identifying viable GCT [34]. Notably, patients in this cohort had negative STMs, further corroborating the importance of miRNA dosing to predict non-teratomatous metastases. Similar results were reported by Leão et al., who observed significantly higher pre-RPLND levels of miR-371a-3p and miR-373- 3p in patients with viable TGCT and a significant decrease in patients with viable GCT after the procedure [35]. Therefore, miRNA could potentially serve as an appealing tool to avoid overtreatment and aid the decision-making process in carefully selected patients.

#### 3.2.2. Chemotherapy

TGCT is regarded as one of the most chemosensitive cancers among all types of malignancies [36]. Thus, chemotherapy represents a key component of TC treatment, particularly for patients with metastatic disease or those at high risk of recurrence after surgery [3]. With a sensitivity of 83.4% and a specificity of 60.1%, miRNAs allowed discriminating between localised and metastasised disease [22,37]. Furthermore, serum miRNA concentration was positively related to disease burden, as patients with more advanced disease had higher miRNA levels [35]. This correlation was further underlined by Dieckmann et al. in a prospective, multicentric study, involving 616 serum samples obtained from TGCT patients. The authors observed a significant decline in terms of miRNA concentrations between the first and second cycles of chemotherapy, regardless of disease stage. On the contrary, a significant reduction between the second and third cycles was achieved only in stage III patients [22]. Hence, the continuous monitoring of miR-371a-3p levels during systemic therapy could offer timely insights into treatment response, even in cases where STMs remain within normal ranges. A predictive role of these non-coding RNA sequences in terms of treatment response has been reported also for chemotherapy. Indeed, baseline levels of miR-371a-3p, miR-373-3p, and miR-367-3p were higher in patients relapsing after a complete response to chemotherapy, compared to patients who achieved a durable response. Furthermore, the average concentration of baseline miR-367-3p was higher in patients who developed refractory disease [32]. Patients with elevated miRNA tended to have intermediate and poor prognoses according to the International Germ Cell Cancer Collaborative Group (IGCCCG) risk groups and experienced worse overall survival and progression-free survival. Nevertheless, statistical significance was lost after adjusting for IGCCCG risk groups [27]. Hence, it is important to recognise miRNAs as indicators of disease burden rather than relapse risk since even patients in subgroups with a less favourable prognosis still exhibit a 50% 5-year overall survival rate. As a matter of fact, their prognostic role in advanced and metastatic TGCTs still needs validation. The main controversies regard the different histological subtypes since only in non-seminoma TGCTs are the pretreatment plasma levels of miR-371a-3p significantly associated with patient outcomes, and no correlation between plasma miR-371a-3p levels and prognostic factors, such as non-pulmonary visceral metastases or extragonadal primary GCTs, was observed [37]. On the contrary, miRNA prognostic relevance emerged in chemo-naïve patients [37] and in patients who were able to achieve complete and durable responses to systemic therapy, with both showing significantly lower pre-chemotherapy concentrations of miRNA [38]. In the precision medicine era, the need for precise biomarkers capable of effectively stratifying patients’ prognostic outcomes and predicting treatment responses underscores the necessity for dedicated research efforts. This requirement will foster the development of prospective trials aimed at resolving uncertainties surrounding the prognostic significance of miRNA.

#### 3.2.3. Active Surveillance and Residual Disease Detection

Given the non-invasive nature of miRNA testing, the most suitable application for these highly sensitive markers seems to be active surveillance and a follow-up of patients treated for TGTCs. This represents a domain where the limited accuracy of STMs has diminished their clinical effectiveness. On the contrary, miR-371a-3p could represent an early marker of relapse after orchiectomy. In a prospective study by Lobo et al., enrolling 151 patients, 94.1% of patients experiencing disease relapse during active surveillance exhibited significantly higher miR-371a-3p levels than post-orchiectomy levels. Notably, only 34.1% in this subgroup had concurrent elevated STMs, and the continuous monitoring of miR-371a-3p levels enabled the earlier detection of relapse compared to STM measurements [39]. This underlines the greater accuracy of miRNA in the early detection of disease recurrence and suggests a turning point in the management of stage I TGCTs, when clinicians grapple with the decision between surveillance and potentially harmful adjuvant therapies, relying solely on clinical criteria. Nevertheless, it must be considered that the accuracy of miR-371a-3p for the detection of disease relapsing is lower (specificity, 96%; sensitivity, 83%) than for the detection of primary TGCT [16]. To define the clinical utility of miRNA in this setting, a panel of four miRNAs (miR371, miR372, miR373, and miR367) will be assessed in a phase III multicentre randomised trial (NCT 03067181) aimed at evaluating the overall survival and progression-free survival of patients undergoing either active surveillance or adjuvant chemotherapy according to disease stage and risk factors [40].

Residual disease detection and monitoring after first-line therapy still represent an unsolved issue, closely tied to final pathology, especially in cases with normal STM levels. Hence, miRNA could cast a new light on disease assessment following orchiectomy and/or systemic therapy, due to their relationship with viable TGCT cells. The predictive value of miRNA in non-seminomatous TGCT was analysed by Leão et al. in the serum samples of 82 patients treated with RPLND. Diagnostic accuracy was remarkable for miR-371a-3p, with an area under the curve (AUC) of 0.874. The addition of miR-373-3p slightly increased the AUC to 0.885. More importantly, sensitivity and NPV both raised to 100% for retroperitoneal lesions >3 cm in diameter [35]. The subsequent findings align with these results, showing that miR-371a-3p was undetectable in patients with fibrosis/necrosis or teratoma [41]. Furthermore, higher miR-371a-3p concentrations were positively related to the extent of residual disease [39]. In this context, a reliable marker for teratoma is lacking, making it challenging to accurately assess treatment success, especially in patients in which traditional imaging after therapy appears negative for residual disease. Indeed, miR-375 has been proposed as a possible biomarker for teratoma, but data about accurateness are still inconclusive. Nappi et al. examined the ability of miR-375, alone or combined with miR-371, to discriminate between teratoma and other histological subtypes. The study enrolled 81 patients either with pathologically confirmed teratoma prior to surgery, clinical stage I TGTC on surveillance, or metastatic seminoma patients prior to chemotherapy. The authors concluded that miR375 was overexpressed in teratoma patients and correlated significantly with residual teratoma burden. Conversely, preoperative miR371 was undetectable in patients with residual teratoma or with complete response after chemotherapy [42]. A high sensitivity (84.2%) in the identification of teratoma with miR-375-5p was reported by Kenigsberg et al. in a prospective study evaluating 40 TGTC patients. However, specificity was limited (35.3%), with a PPV and NPV of 59.3% and 66.7%, respectively [43]. Nevertheless, evidence that rejects the role of miR-375 was outlined by Lobo et al. and Berge et al. in two retrospective studies on serum samples of patients suffering from TGTC. In both cases, no significant difference was observed in terms of serum levels of miR-375 between teratoma, necrotic/fibrotic tissue, and other histological subtypes [26,44]. Discrepancies between these results could be explained by differences in terms of study design, sample sizes, and methodological issues (i.e., collecting tubes and samples used to analyse miRNA concentrations). Although certain outcomes might suggest the clinical utility of miR-375 as a predictor of components of residual TGTC, the performance characteristics of miR-375 as a standalone biomarker appeared modest. Therefore, further validation is imperative before considering its routine application in clinical practice. Furthermore, despite the promising improvement in predictive accuracy for teratoma when miR-371 was combined with miR-375, partially mitigating the limitations associated with miR-375 alone, it is essential to note that contradictory findings regarding miR-371 exist in the available literature, as outlined earlier in this review (Table 1).

## 4. Future Perspective

MiRNAs have the potential to answer open clinical questions in the management of TGTC. Nonetheless, certain aspects regarding their current applicability still need to be addressed. From a technical standpoint, universally accepted guidelines are needed to streamline the methodology for isolating, detecting, quantifying, and reporting miRNA levels. These protocols should encompass a variety of bodily samples (i.e., plasma and serum) and should address the normalisation methods and the determination of appropriate cut-off values. This standardisation is essential to ensure the reliability and comparability of measurements across different research studies and clinical settings [26]. Moreover, some aspects pertaining to the clinical utility of these non-coding RNA sequences in TGTC still hamper their widespread use, namely, their prognostic value and their accuracy in detecting residual disease. Several ongoing prospective trials aim to exhaustively answer these open questions. Besides the aforementioned randomised trial conducted by the Children Oncology Group, a prospective observational cohort study is currently recruiting patients in order to estimate the PPV of miR-371 in detecting germ cell malignancies within both early stage testicular seminoma and non-seminoma groups, using plasma expression levels at the time of relapse [45]. The clinical importance of a reliable marker for TGTCs is compelling, especially in some clinical scenarios, such as the early stages, in which even the odds of selecting the correct treatment strategy exist. The CLIMATE trial is an ongoing prospective, single-arm cohort study designed to compare the relapse-free survival among miR-371 positive and negative clinical stage I TGCT patients undergoing active surveillance [46]. The early detection capability of miRNA, before radiographic or biochemical evidence, could present notable advantages. The need for surveillance imaging could diminish, thereby reducing exposure to ionizing radiation from CT scans and alleviating treatment uncertainty in patients undergoing active surveillance. More importantly, it opens the door for timely intervention in relapse cases, potentially leading to reduced morbidity compared to the conventional approach of waiting for clinical relapse detection [26]. Nevertheless, the implementation of novel biomarkers must consider the cost-effectiveness of the development process and their impact on the healthcare system [27]. The cost-effectiveness of a miRNA-based approach was demonstrated using a decision tree model in patients with stage I non-seminomatous TGTCs. Therapeutic approaches guided by miRNA levels were less expensive than the standard decision-making process by ~USD 1400 per patient. Moreover, the rates of treatment appropriateness and reduction in overtreatment and undertreatment favoured the miRNA-based approach [47]. 

The utilisation of miRNAs holds significant promise in solving many uncertainties surrounding the diagnosis and therapeutic management of testicular neoplasia. This emerging field could transform our understanding of testicular cancer and provide more precise tools for early detection and treatment stratification. Notwithstanding the significant progress achieved in this field, it is essential to acknowledge certain existing challenges. One of the primary hurdles is the lack of prospective, randomised clinical trials assessing the effectiveness of microRNA-based approaches in TGTCs. These are imperative for establishing the utility, sensitivity, and specificity of microRNA markers, as well as their integration into clinical practice. Additionally, the limited availability of long-term follow-up data hampers our ability to draw definitive conclusions about the clinical benefits and outcomes associated with microRNA-based diagnostic and therapeutic strategies. 

In summary, while microRNA-based techniques represent a burgeoning frontier in the field of TC, their full potential can only be realised through the establishment of well-designed clinical trials and the accumulation of comprehensive long-term data, ensuring their translation into effective clinical tools. 

## 5. Strengths and Limitations

The present study represents an extensive and thorough review of the current state of knowledge regarding miRNAs in TGCTs. It covers different aspects of this topic, including the utility of miRNAs as diagnostic tools and prognostic markers, as well as their role in guiding treatment decisions, effectively summarizing available literature and providing a complete understanding of the subject. Moreover, it focuses on the clinical implications of miRNAs in TGCTs, offering clinicians a valuable tool for the clinical decision-making process in daily practice. Despite its strengths, some limitations must be acknowledged when interpreting our results. First, the lack of a systematic approach in this study may introduce subjectivity and hinder reproducibility. While the authors’ expertise in selecting the included studies helps mitigate this limitation, it remains challenging to completely eliminate heterogeneity in study selection. Moreover, heterogeneity in terms of design and clinical outcomes among the included study could hamper the generalisability of our results. As a result, synthesizing the evidence in a manner that allows for clear conclusions and recommendations may be difficult for the reader. Moreover, the absence of prospective randomised trials lowers the quality of evidence that can be collected and conveyed in the present review. Nevertheless, prospective trials are currently underway with the aim of filling existing gaps about the clinical utility of miRNAs in TGCT management and the potential cost-effectiveness of miRNA-based approaches. These forthcoming trials promise higher quality and more reliable evidence. The supplement of additional evidence from well-designed clinical trials and long-term data will undoubtedly facilitate the definitive validation of the clinical utility of miRNAs in the treatment of TGCTs. The subject matter of this manuscript is undoubtedly complex, and its utility in daily practice may be a subject of debate. Nevertheless, we strongly contend that more sensitive biomarkers for tailoring the management of TGCT patients are both compelling and represent a fundamental resource for clinicians.

## 6. Conclusions

In conclusion, TGCTs still pose significant challenges in diagnosis and treatment. While conventional serum tumour markers and clinical assessments have intrinsic limitations, miRNAs have emerged as promising biomarkers with the potential to overcome these challenges. In the realm of diagnostics and disease management, miR-371a-3p has demonstrated its potential as a sensitive and specific biomarker for TGCT, showing high sensitivity and specificity values in several studies. Moreover, the existing relationship between miRNA concentration and response to treatment across different disease stages confers the potential for treatment response monitoring and predicting disease relapse. Still, ongoing clinical trials are dedicated to addressing existing issues concerning the clinical utility of miRNA in TGCT. Additionally, the standardisation of some technical aspects concerning miRNA and their cost-effectiveness and impact on the healthcare system must be carefully considered. Hence, while challenges and uncertainties remain, future research and ongoing clinical trials are essential to harness the full potential of miRNAs in these critical aspects of TGCT care.

## Figures and Tables

**Table 1:** miRNA function in testicular cancer.

miRNA	Detection Site	Expression	Role	Clinical Function
miR-302 [13,14,15]	Plasma GCNIS and TGCT tissues	↑		
miR-367-3p [13,14,15,32]	Plasma GCNIS and TGCT tissues	↑	Diagnosis Prognosis	Higher in patients relapsing after chemotherapy/refractory disease
miR-302abc/200c [23]	Plasma	↑	Diagnosis	
miR-371-3/367 [23]	Plasma	↑	Diagnosis	
miR-371-3 [27]	Plasma GCNIS and TGCT tissues	↑ or ↓	Tumour size	
miR-371a-3p [32,35,41]	Plasma and hydrocele fluid of patients with TGCT	↑ or ↓	Diagnosis Prognosis	Increases in presence of GCT/residual disease Decreases after radical orchiectomy/chemotherapy Higher in patients relapsing after chemotherapy
miR-372 [31,32,33]	Plasma	↓	Prognosis	Decreased levels in response to chemotherapy Disease relapse
miR-373-3p [32,35]	Plasma	↑	Prognosis	Higher in patients relapsing after chemotherapy
miR-375 [42,43,44]	Plasma	↑	Diagnosis	Possible biomarker for teratoma (combined with miR-371) Higher in patients with residual disease
miR-371 [45,46]	Plasma	↓	Diagnosis	Possible biomarker for teratoma (combined with miR-375)

## Data Availability

Data are available in scientific databases.
